# The elite judo female athlete’s heart

**DOI:** 10.3389/fphys.2022.990142

**Published:** 2022-08-26

**Authors:** Aleksandra Milovančev, Milovan Petrović, Tatjana Miljković, Aleksandra Ilić, Tatjana Redžek Mudrinić, Aleksandar Miljković, Olivera Ivanov, Jelena Tripunović, Bogdan Anđelic, Antonino Bianco, Patrik Drid

**Affiliations:** ^1^ Faculty of Medicine, University of Novi Sad, Novi Sad, Serbia; ^2^ Institute of Cardiovascular Diseases of Vojvodina, Sremska Kamenica, Serbia; ^3^ Sport and Exercise Sciences Research Unit, University of Palermo, Palermo, Italy; ^4^ Faculty of Sport and Physical Education, University of Novi Sad, Novi Sad, Serbia

**Keywords:** combat sports, physiological adaptation, echocardiography, left ventricular geometry, ventricular remodeling

## Abstract

**Purpose:** There is a paucity of data on physiological heart adaptation in elite-level judo female athletes. This study aimed to assess left ventricular morphology and function in highly trained elite female judokas.

**Methods:** The study prospectively included 18 females aged 23.5 ± 2.25 years, nine elite level judokas, and nine healthy non-athlete volunteers. All participants underwent a medical examination, electrocardiogram, and transthoracic 2D echocardiogram. Left ventricular diastolic and systolic diameters and volumes were determined, and parameters of left heart geometry and function (systolic and diastolic) were measured, calculated, and compared between groups.

**Results:** When groups were compared, judokas had significantly increased left ventricular cavity dimensions *p* < 0.01, left ventricular wall thickness *p* < 0.01, and volumes *p* < 0.01. Elite female judokas exhibited left ventricular dilatation demonstrated as high prevalence increased end-diastolic volume/index, and increased end-systolic volume/index in 88.9% of judokas vs. 0% in controls, *p* < 0.01. Left ventricle mass/index was significantly increased in judokas, *p* < 0.01), with a 43.3% difference between groups. The majority (77.7%) of judokas had normal left ventricular geometry, although eccentric hypertrophy was revealed in 2 (22.2%) of judokas.

**Conclusion:** Elite, highly trained female judokas exhibit significant changes in left heart morphology as a result of vigorous training compared to non-athletes. These findings suggest that female judokas athletes’ heart follows a pattern toward chamber dilatation rather than left ventricular wall hypertrophy.

## Introduction

Judo is a popular combat sport worldwide. As reports from International Judo Federation (IJF) claim, there are 1,181 female judokas competing on an elite level, spread across all weight categories. Despite many proven positive physiological benefits of practicing judo, it inevitably leads to the exhibition of various adaptations of the cardiovascular system (Drid et al., 2021). Combat sports are characterized by the multifactorial influence of dynamic and high-intensity activities; nevertheless, the unique volume of work and energy demands in judo during the training period (i.e., preparation phase) and match differentiate. Physiological heart adaptations are affected by exercise workload and are closely related to the type of sport (Whyte et al., 2000; Degoutte et al., 2003).

Over time these complex adaptive changes usually lead to the development of the “athlete’s heart” (Maron and Pelliccia, 2006; Pelà et al., 2016). Early reports on athletes’ heart characteristics were based on male athletes, and findings suggested that endurance sports follow a pattern of ventricular dilatation and eccentric hypertrophy, while resistance sport disciplines result in ventricular wall hypertrophy and concentric remodeling (Morganroth et al., 1975). Later studies reported that cardiac remodeling is dependent on sex (Pelà et al., 2016) and sports discipline (Whyte et al., 2000). But still, the majority of studies dealing with this topic, especially those with larger samples, excluded female athletes (Pluim et al., 2000). Hence, female athletes’ heart is less frequently studied with limited data, mainly reporting that women have a relatively larger increase in cavity dimensions compared with men (D’Ascenzi et al., 2020; Whyte et al., 2004). The studies pertaining to female athletes’ heart characteristics had a low prevalence of judokas, and results were interpreted usually in the context of resistance sports disciplines (Pelliccia et al., 1996; Sun et al., 2007). Whyte et al. performed a study exclusively on judokas and found that left ventricular remodeling was similar between sexes with increased ventricular thickness and left ventricular mass compared to matched controls (Whyte et al., 2000) in contrary to Laskowski et al. who reported that heart adaptations such as the increased diastolic dimension of the left ventricle in elite male and female judoists resemble adaptations observed in endurance athletes (Laskowski et al., 2008).

Understanding the specific features of the athlete’s heart in specific populations is of great importance in order to know what to expect of a specific athlete’s sports discipline and gender. Initial and follow-up examinations help us to differentiate physiological exercise that induces cardiac remodeling from pathological conditions. Differentiating exercise-induced cardiac remodeling of abnormal cardiovascular pathology is important in screening and diagnosing potentially life-threatening conditions. Essentially, there is limited data regarding female athletes’ heart response to long-term elite-level judo participation and training stimulus (Laskowski et al., 2008; Whyte et al., 2000). To our best knowledge, there are no studies evaluating cardiac adaptations exclusively, only elite female judokas, and further investigation in the field is needed.

This study aimed to assess left ventricular morphology and function in highly trained elite female judokas.

## Materials and methods

### Participants

The study prospectively included 18 females aged 23.5 ± 2.25 years. The Judo group consisted of nine elite-level judo athletes (i.e., judokas) and nine healthy non-athlete female student volunteers previously not involved in regular exercise training for the last 5 years in the control group. All judokas were a part of the national team, participating in both national and international contests. Subjects started with judo practicing between six and 8 years old, the average duration of the judo training experience was 16.55 ± 3.16 years, with twenty to twenty-five (20-25) hours of training volume per week. Judokas spent 1,5-2 h of training in the weight room two to three times a week. The participants competed for the Serbia senior national team in international competitions and scored for the IJF rank, and they achieved the minimum of one ranking. Two competitors were category up to 48 kg, two −52, one −57, two −63, one −78, and one +78 kg. They are all still competing. Four of them achieved the Olympics norm (the 10th, 10th, 16th, and 18th place WRL), and two were close to the Olympics norm and the 25th place. Three of them participated in the Olympics, one didn’t because two were in the same category −48 kg, but only one could participate.

All participants underwent a medical examination, electrocardiogram (ECG), and transthoracic 2D echocardiogram at the Institute for Cardiovascular Diseases of Vojvodina, Serbia. All participants underwent a standard resting 12-lead surface ECG record at a paper speed of 25 mm/s and gain of 10 mm/mV. One cardiologist interpreted each ECG to exclude potential abnormalities. Exclusion criteria were: A previous history of arterial hypertension, diabetes mellitus, heart, kidney, hepatic, infectious, psychiatric, malignant, disorders, taking drugs that can influence ECG intervals, and electrolyte imbalance. The study was performed corresponding to the Helsinki Declaration with ethical approval obtained from the local ethics committee of the University of Novi Sad, Serbia (Ref. No. 46-06-02/2020-1). Measurements were done in the early morning hours before any water or food intake. For the judo group at least 24 h after the last training. Body height (BH) measurement was performed in a standing position with heels together, toes apart, and hands close to the body with an anthropometer, according to Martin (GPM, Switzerland), with an accuracy of 0.1 cm. Body mass (BM) was determined using an Omron weight scale BF511 (Omron, Osaka, Japan) with an accuracy of 0.1 kg. Body surface area (BSA) and body mass index (BMI) were calculated using the following formulas:

$$\text{BMI }={\text{ BW/ BH}}^{2}\;\left(\text{BMI Calculator, n}\text{.d}\text{.}\right)\text{ and BSA }\left(\text{m}2\right)=\frac{\sqrt{\text{BH }\left(\text{cm}\right)}\text{ x BW }\left(\text{kg}\right)\;}{3600}$$

(Du Bois and Du Bois, 1916).

### Echocardiography

All subjects underwent two-dimensional transthoracic echocardiography (ECHO) using Vivid E9 (GE Healthcare, Milwaukee, WI, United States) machine that is equipped with an M5S-D, 1.5–4.6 MHz transducer with continuous ECG monitoring during the examination. Subjects were instructed to take a left lateral decubital position for examination. One investigator, blinded to the clinical characteristics of the participants, without blinding was evaluating echocardiographic parameters (for each acquisition, three cardiac cycles of uncompressed data were stored in cine-loop format). All measurements and calculations were performed by previously described methods according to standardized procedures by the European Association of Cardiovascular Imaging (Lang et al., 2005) and the American Society for echocardiography, and the European Association of Cardiovascular Imaging. Different parameters were measured and compared.

Parasternal long-axis view (2D) was used to measure LV end-diastolic (LVEDd) and systolic (LVEDs) diameter, the diastolic thickness of the inter-ventricular septum (IVS), posterolateral wall (PLW), aortic root, ascending aorta dimension and cusp separation. Volumes of the left atrium (LA) were measured by the Simpson method using apical 4-chamber and 2-chamber views at end-systole in maximum LA size (LAVs) and then normalized for body surface area (BSA) as LAVs index (LAVsI). Using the biplane method of disks, left ventricular end-diastolic (EDV) and end-systolic volumes (ESV) were measured, and EF was calculated. Left ventricular mass (LVM) was calculated by the software automatically via area–length method, according to measures obtained from parasternal short-axis view in which mid-ventricular systolic and diastolic epicardial and endocardial surfaces were traced (with the exclusion of papillary muscles), including measuring the systolic and diastolic mitral-to-apical distance in apical 4-chamber view. LVM = 1, 05 (5/6A2 (L + *t*)–(5/6A2L), where A1 is epicardial area at end-diastole (cm2), A2 endocardial area at end-diastole (cm2), L-ventricular length at end-diastole (cm), t = average wall thickness (cm), 1, 05-specific gravity of the muscle (g/ml). To obtain the indexed value (LVMi) the LVM was divided by BSA. Calculation of left ventricular hypertrophy level (LVHL) was calculated by adding intraventricular septum diameter (IVSDd) and posterior wall thickness (PLWd) and dividing the sum by two (LVHL= (IVSDd + PLWd)/2).

According to left ventricular mass index (LVMI) and relative wall thickness (RWT), left ventricular morphology is classified into one of four structural groups: 1) normal geometry, characterized by normal LVMI ≤95 g/m2 and RWT ≤42 mm; 2) concentric remodeling, LVMI ≤95 g/m2 and RWT >42 mm; 3) Concentric hypertrophy, LVMI>95 g/m2 and RWT>42 mm; and 4) Eccentric hypertrophy LVMI>95 g/m2 and RWT ≤42 mm (8).

In the apical 4-chamber view, using transmitral pulsed-wave (PW) Doppler at the tips of mitral leaflets peak early wave (E), atrial late (A) diastolic filling velocities were measured, and E/A ratio was calculated. Tissue Doppler on the mitral annulus was obtained at the septal and lateral positions, where peak early (e′) velocities were measured and averaged. E/e' ratio was calculated from E velocity and averaged e' velocities obtained from septal and lateral positions of the mitral annulus.

### Statistical analysis

The analysis included descriptive statistics. To test the normal distribution, the Kolmogorov-Smirnov test was used. Continuous variables data are presented as mean ± standard deviation. Categorical variables are expressed as absolute numbers and percentages. The data were compared using the Students *t*-test or Chi-square test where appropriate. A *t*-test was used to compare continuous variables with normal distribution, and a chi-square test was used to compare categorical variables. A *p*-value < 0.05 was considered statistically significant. All calculations and interpretations were made using Statistical Package for Social Sciences–SPSS Version 20.0 (IBM Corp. 20, Armonk, NY).

## Results

There was no statistically significant difference between judo and control group for: BH 166.56 ± 7.29 vs. 167.44 ± 10.78 cm, *p* = 0.84, BW 63.92 ± 13.87 vs. 62.67 ± 9.35 kg, *p* = 0.83, BMI 22.82 ± 6.64 vs. 22.31 ± 3.04 (kg/m2) *p* = 0.83, and BSA 1.70 ± 0.19 m^2^ vs. 1.71 ± 0.18 m^2^, *p* = 0.93. The mean age of judo athletes was 24.67 ± 3.84 years, while the mean age of the control group was 23.44 ± 0.88 years, *p* = 0.38.

When groups were compared judokas had significantly increased left ventricular cavity dimensions (LVEDd 49.06 ± 2.79 vs. 43.11 ± 2.03 mm, *p* < 0.01, LVEDd/BSA 29.05 ± 2.34 vs. 25.45 ± 2.56 mm/m2, *p* < 0.01, with 22.46% difference, LVEDs 33.11 ± 2.32 vs. 27.00 ± 2.55 mm, *p* < 0.01, and LVEDs/BSA 19.63 ± 2.04 vs. 16.03 ± 2.58 mm/m2, *p* < 0.01, with 14.14% difference), LV wall thickness (IVS thickness 8.42 ± 0.51 vs. 7.60 ± 0.71 mm, *p* < 0.01 with 17% of difference and PW thickness 8.48 ± 0.63 vs. 7.30 ± 0.47 mm, *p* < 0.01 with 16% of difference) and volumes EDVI 70.07 ± 7.75 vs. 49.12 ± 6.32ml/m2, *p* < 0.01, ESVI 28.39 ± 3.14 vs. 17.15 ± 2.40 ml/m2, *p* < 0.01, (Table 1). Judokas in 11.1% exhibited abnormal LVEDs/BSA as mildly increased diameter above 22mm/m2, significantly increased EDV (>106 ml) and EDVI (>61 ml/m2) in 88.9% vs. 0%, *p* < 0.01 in controls and increased ESV (>42 ml) and ESVI (>24 ml/m2) in 88.9% vs. 0% of controls, *p* < 0.01.

**TABLE 1:** Echocardiographic characteristics of the study population.

Parameter	Judo *n* = 9	Control *n* = 9	p
	Mean ± SD	Mean ± SD	
Left ventricle			
LVEDd (mm)	49.06 ± 2.79	43.11 ± 2.03	<0.01
LVEDd/BSA (mm/m^2^)	29.05 ± 2.34	25.45 ± 2.56	0.01
LVEDs (mm)	33.11 ± 2.32	27.00 ± 2.55	<0.01
LVEDs/BSA (mm/m^2^)	19.63 ± 2.04	16.03 ± 2.58	<0.01
IVS (mm)	8.42 ± 0.51	7.60 ± 0.71	0.01
PLW (mm)	8.48 ± 0.63	7.30 ± 0.47	<0.01
EDV (ml)	118.67 ± 13.85	83.33 ± 8.73	<0.01
EDVI (ml/m^2^)	70.07 ± 7.75	49.12 ± 6.32	<0.01
ESV (ml)	46.44 ± 6.35	29.85 ± 3.89	<0.01
ESVI (ml/m^2^)	28.39 ± 3.14	17.15 ± 2.40	<0.01
SV (ml)	72.22 ± 8.73	54.28 ± 6.15	<0.01
SVI (ml/m^2^)	42.60 ± 4.35	31.17 ± 3.91	<0.01
LV geometry			
LVHL	8.45 ± 0.55	7.45 ± 0.54	<0.01
RWT (mm)	0.35 ± 0.02	0.34 ± 0.03	0.67
LVM (g)	143.09 ± 21.93	100.00 ± 5.87	<0.01
LVMI (g/m^2^)	84.67 ± 14.06	59.10 ± 6.83	<0.01
Aortic dimensions			
AORTIC ROOT (mm)	23.78 ± 1.72	20.89 ± 0.93	<0.01
CUSPIS SEPARATION (mm)	20.67 ± 1.80	17.67 ± 1.32	0.01
ASCENDING AORTA (mm)	27.22 ± 1.86	26.67 ± 1.97	0.58
LV systolic and diastolic function			
EF%	60.89 ± 2.71	64.78 ± 1.56	<0.01
FS%	32.50 ± 2.86	37.27 ± 6.40	0.07
E–wave (m/s)	0.82 ± 0.13	0.92 ± 0.12	0.09
A (m/s)	0.45 ± 0.10	0.57 ± 0.09	0.02
E/A ratio	1.87 ± 0.41	1.67 ± 0.38	0.29
e’ sep (cm/s)	0.13 ± 0.01	0.13 ± 0.02	0.74
E/e’sep ratio	6.29 ± 1.01	6.83 ± 1.34	0.37
e’ lat (cm/s)	0.19 ± 0.02	0.15 ± 0.03	0.01
E/e’ lat ratio	4.37 ± 0.84	6.28 ± 1.50	<0.01
e’average (cm/s)	0.16 ± 0.15	0.14 ± 0.02	0.07
E/e’ average	5.14 ± 0.84	6.59 ± 1.28	0.01
LAVs (ml)	47.22 ± 8.36	36.89 ± 4.11	0.04
LAVsI (ml/m2)	27.78 ± 4.22	21.74 ± 2.74	0.02

A-late atrial contraction, E-early wave, E/A ratio–peak early wave to atrial late wave ratio, e’average -average peak early velocity, E/e’ average-early wave to average peak early velocity, E/e’ sep-early wave to septal peak early velocity, E/e’ lat ratio-early wave to lateral peak early velocity, e’ lat-lateral peak early velocity, e’ sep-septal peak early velocity, E/e’ lat ratio-early wave to lateral peak early velocity, EDV-LV, End-diastolic volume; EDVI- LV, End-diastolic volume/body surface area, EF- ejection fraction % ESV–LV, End-systolic volume; ESVI–LV, End-systolic volume/body surface area; FS, fraction of shortening %, IVS, inter-ventricular septum, LVHL–left ventricle hypertrophy level, LAVs–left atrium volume at end-systole, LAVsI–left atrium volume index; LVM–left ventricle mass; LVMI–left ventricle mass index, LVEDd–LV, end-diastolic diameter, LVEDd/BSA–LV, end-diastolic diameter and body surface area ratio; LVEDs–LV, end-systolic diameter, LVEDs/BSA–LV, end-systolic diameter and body surface area ratio, p–statistical significance (p < 0.05), PLW, posterolateral wall, RWT–relative wall thickness, SD -standard deviation, SV–Stroke volume, SVI–stroke volume/body surface area.

**FIGURE 1:**
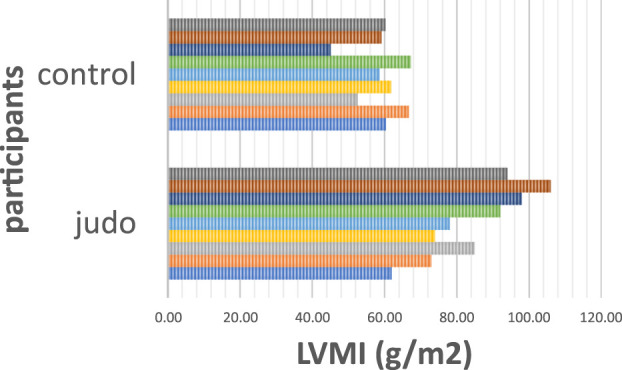
Comparison of values of LVMI between elite female judokas and non-athletes.

The difference in LVHL among the judo and controls group was statistically significant 8.45 ± 0.55 vs. 7.45 ± 0.54 mm, *p* < 0.01, as well as LVM 143.09 ± 21.93 vs. 100.00 ± 5.87 g, *p* < 0.01 and LVMI 84.67 ± 14.06 vs. 59.10 ± 6.83 g/m2, *p* < 0.01, (Figure 1), which amounted to a 43.3% difference between groups. Judokas had mildly increased LVM (>162 g) and LVMI (>95 g/m2) in 22.2% vs. 0% of controls, *p* = 0.14.

Majority of judokas (77.7% vs. 100% of non-athletes, *p* = 0.14) exhibited normal left ventricular geometry. Eccentric hypertrophy was found in two judokas (who had an Olympic norm) 22.2% vs. 0%, *p* = 0.14.

Aortic dimensions in root 23.78 ± 1.72 vs. 20.89 ± 0.93 mm, *p* < 0.01, and cusp separation were significantly higher in judokas, 20.67 ± 1.80 vs. 17.67 ± 1.32 mm, *p* = 0.01, respectively.

Both groups remained within the normal systolic and diastolic function range. We observed a statistically significant difference in systolic function, the mean EF in judokas was 60.89 ± 2.71 vs. 64.78 ± 1.56 in controls, *p* < 0.01. Stroke volume/index was significantly higher in judo athletes, *p* < 0.01. Significant differences were observed in several parameters of diastolic function: A wave in judokas 0.45 ± 0.10 vs. 0.57 ± 0.09, *p* = 0.02, e’ lat 0.19 ± 0.02 vs. 0.15 ± 0.03, *p* = 0.01, E/e’ lat 4.37 ± 0.84 vs. 6.28 ± 1.50, *p* < 0.01, E/average 5.14 ± 0.84 vs. 6.59 ± 1.28, *p* = 0.01, respectively. Judokas exhibited significantly higher volumes of left atrium, LAVs 47.22 ± 8.36 vs. 36.89 ± 4.11, *p* = 0.04, LAVsI 27.78 ± 4.22 vs. 21.74 ± 2.74, *p* = 0.02.

## Discussion

The study showed that elite female judokas exhibit significant physiologic changes in left heart morphology and function compared to non-athletes. In elite female judokas, systolic, diastolic diameters, volumes, and wall thickness of the left ventricle were significantly increased compared to controls. Additionally, the female judo group observed a high prevalence of 88.9% of abnormally increased left ventricular end-diastolic and end-systolic volumes. There were no abnormal values of left ventricular diastolic diameters or wall thickness.

A Pioneer study (Morganroth et al., 1975) on 42 male athletes compared to 12 controls revealed that isometric (strength) sports exhibited greater left ventricular wall hypertrophy equivalent to concentric remodeling/hypertrophy, while isotonic (endurance, dynamic) sports exhibited greater left ventricular volumes equal to eccentric hypertrophy. Morganroth’s hypothesis proposed that endurance exercise sports result in eccentric hypertrophy, while power/resistance sports result in concentric hypertrophy. But this hypothesis has been poorly tested on female athletes. According to types of exercises, judo is classified as a high static and low dynamic sport, requiring high levels of intramuscular force (Mitchell et al., 1994), thus producing an intermittent but considerable hemodynamic pressure overload (MacDougall et al., 1985). We would expect, based on Morganroth’s hypothesis, elite judokas have predominantly concentric remodeling/hypertrophy. Nevertheless, on the contrary, the female judo athletes in our study exhibit normal diameters but increased volumes above the reference range in high prevalence (volumetric assessment is more precise than the diameter for determining left ventricular size). The ventricular wall thickness was increased compared to controls but not above reference values.

The “Morganroth hypothesis” has been widely adopted in professional literature, partly as a result of a large group of cross-sectional proof that suggests endurance athletes tend to have increased cavity dimensions when compared to control subjects or resistance sports athletes (Naylor et al., 2008). However, in a meta-analysis that encompassed 59 studies and 1,451 men athletes (Pluim et al., 2000), the authors concluded that endurance sports training is associated with both left ventricular internal diameter dilatation and an increase in left ventricular wall thickness. Strength training hearts showed a large increase in LV thickness and a slight increase in LV internal diameters. Further, disciplines with a combination of strength and endurance exhibit the largest increases in left ventricular internal dimension and left ventricular wall thickness. Authors suggested that remodeling is more complex than first thought, and it depends on various factors.

Only limited evidence of cardiac adaptation to resistance training in females has been published. An early cross-sectional report (George et al., 1998) that included 24 female weightlifters demonstrated a concentric left ventricular enlargement. In a unique study on 600 highly trained women athletes, Pelliccia et al. (Pelliccia et al., 1996) demonstrated dimensional cardiac changes but without substantial increases in absolute LV wall thickness, which was within normal limits for all women athletes. Only 13 athletes in this study were elite-level female judokas. They reported results of LVMI of 81 ± 17 g/m2 and LVEDd of 48 ± 4.9 mm (LVMI 84.67 ± 14.06 g/m2, LVEDd 49.06 ± 2.79 mm), which seems well supportive of our findings.

In a study on 174 female Chinese athletes (4.7% of judokas), left ventricular wall thickness didn’t exceed 11mm, but 10.3% exhibited left ventricular internal diameter above 50 mm, and 4.2% above 55 mm (Sun et al., 2007). In our study, 33.3% of athletes had LVEDd above 50 mm, contrary to other findings (Whyte et al., 2000), where 17 female judokas demonstrated significantly increased wall thickness and LVM compared to matched controls and suggested that cardiac adaptation in females resembles those in males. Concomitantly, similar mean values are noticeable among judokas in both studies. Our results are comparable to Laskowski et al., who reported that increased LV dimensions resemble changes observed in endurance athletes in a study that included elite-level judokas (20 males and 15 females) (Laskowski et al., 2008). Aortic dimensions were normal in both groups, as suggested by numerous authors (Maron, 2009; D'Andrea et al., 2010; Pelliccia et al., 2010; Boraita et al., 2016; Gati et al., 2019; Churchill et al., 2020). Although there were differences between athletes and controls, systolic and diastolic functions were preserved among all participants. Whyte et al. found higher values of EF among female judokas. Still, they remained within the range of normal values as well as other authors reported (Pelliccia et al., 1996; Whyte et al., 2004; Sun et al., 2007). We also observed significant differences in left atrial volumes between groups but without abnormal values. Left atrium volume is poorly studied in female athletes, and its dilatation may reflect increased preload. This is considered a part of the physiological remodeling process of long-term endurance exercise (Prior and la Gerche, 2012).

Our results confirm that athlete training is associated with enhanced systolic and diastolic function, probably mediated by a combination of enhanced early relaxation and increased LV compliance. The diastolic function shows evidence of enhancement in lower late atrial A wave, higher e' waves, and lower filing velocities E/e.’ Greater stroke volumes and SVI reflect improvement in systolic function. In a large meta-analysis, when groups of endurance-, resistance- and mixed-training athletes and healthy controls were compared, the E/A ratio was either normal or slightly, but not significantly, enhanced in athletes compared to controls (Pluim et al., 2000). In 1991 group of authors found that endurance-trained athletes exhibited greater stroke volumes than controls at the same pulmonary capillary wedge pressure (Levine et al., 1991) and suggested that endurance training improved ventricular compliance. Additional results (Stickland et al., 2006) confirmed that left ventricular filling pressures were lower in athletes than controls at similar submaximal stroke volume.

Whyte et al., in a meta-analysis of 13 studies (890 female athletes and 333 non-athletes), including 38 (16.7%) judokas (classified into the strength and sprint group) suggested that female athletes follow a similar pattern of cardiac adaptation as men (Whyte et al., 2004). As there is no data reported exclusively on judokas drawing a conclusion only based on sports discipline may be misleading. We believe that an individual approach (with sport type, gender, and level of training as some of the most important parameters) is needed when distinguishing the type of physiological remodeling of a pathological condition. Contrary to results reported by Whyte et al., several previous studies stated that cardiac remodeling is gender-specific (Pelliccia et al., 1996; Pelà et al., 2016).

Finocchiaro et al. showed that 71% of highly trained female athletes generally show normal LV geometry (Finocchiaro et al., 2017). Our study confirmed previous findings where 78% of female judokas exhibit normal geometry. Eccentric LV hypertrophy (22%) was the most common physiological response in females involved in endurance sports (compared with 16% of males; *p* < 0.001) (Finocchiaro et al., 2017), these findings are comparable to our results where 22.2% had eccentric hypertrophy. The cardiac adaptation pattern in elite female judokas rather resembles one in endurance sports. Judo is a combat sport of short duration where the fight lasts approximately 7.18 min (Torres-Luque et al., 2016), 3–4 min, or only several seconds (Franchini et al., 2011). We hypothesize that judo, due to intensive intermittent workload (Krstulović, 2012; Krstulović and Sekulić, 2013) prior to and during the match, should be classified as high static activity (Mitchell et al., 1994). Echocardiography is a valuable diagnostic technique that allows a comprehensive initial evaluation of cardiac structures, function, and response to exercise to distinguish normal and physiological from pathology (Grazioli et al., 2015). These results have important clinical implications for physicians involved in initial and follow-up examinations of elite female judo athletes. Cardiac remodeling in this specific population could be mainly due to left ventricular enlargement and not to increased wall thickness, as should be expected, with judo being categorized as a strength sport. Elite judokas follow a pattern of exercise-induced eccentric hypertrophy remodeling, which is usually expected in endurance sports.

These findings confirm that an athlete’s physiological cardiac adaptation pattern is much more complicated than first thought and that it can’t be generalized to a sports discipline. It probably depends on numerous individual parameters like individual sport type, duration of practicing, weekly training volume, the athlete’s weight category, and other variables that influence the physiology of heart adaptation. Further longitudinal studies could help us piece the puzzle together and see the whole picture.

### Limitations

Admittedly, the first weakness of this study is the small sample size of only *n* = 18 subjects. However, our sample consisted of nine elite-level judokas competing at an international level, where four of our subjects met entry norms for the Olympic games in 2020. Therefore, recruiting a larger number of athletes at this level is constricted. The measurements were only performed once by only one echocardiographist, and parameters are only indexed to BSA, which may not represent the most precise method.

## Conclusion

These findings suggest that elite, highly trained female judokas exhibit significant changes in left heart morphology. Furthermore, athletes’ heart follows a pattern toward chamber dilatation rather than left ventricular wall hypertrophy. The study deals with the population of judo elite-female athletes that have not been exclusively previously investigated, furthermore gives us a different insight into the cardiac remodeling in this specific population. These results indicate that cardiac remodeling is complex and depends on undouble different factors. The findings might assist us in perceiving physiological training-induced adaptations from pathological conditions which may predispose adverse cardiovascular events among athletes. Results may encourage us for further research to strengthen our knowledge related to specific heart structure and function adaptations that play a crucial role in heart remodeling classification and the distinction between pathophysiology and individual sport-specific adaptations.

## Data Availability

The raw data supporting the conclusion of this article will be made available by the authors, without undue reservation.
